# Significant impact of amount of PCR input templates on various PCR-based DNA methylation analysis and countermeasure

**DOI:** 10.18632/oncotarget.10906

**Published:** 2016-07-28

**Authors:** Zhaojun Liu, Jing Zhou, Liankun Gu, Dajun Deng

**Affiliations:** ^1^ Key Laboratory of Carcinogenesis and Translational Research (Ministry of Education/Beijing), Division of Etiology, Peking University Cancer Hospital and Institute, Haidian District, Beijing, 100142, China

**Keywords:** DNA methylation, false negative detection, MethyLight, DHPLC, MSP

## Abstract

Methylation changes of CpG islands can be determined using PCR-based assays. However, the exact impact of the amount of input templates (TAIT) on DNA methylation analysis has not been previously recognized. Using *COL2A1* gene as an input reference, TAIT difference between human tissues with methylation-positive and −negative detection was calculated for two representative genes *GFRA1* and *P16*. Results revealed that TAIT in *GFRA1* methylation-positive frozen samples (*n* = 332) was significantly higher than the methylation-negative ones (*n* = 44) (*P* < 0.001). Similar difference was found in P16 methylation analysis. The TAIT-related effect was also observed in methylation-specific PCR (MSP) and denatured high performance liquid chromatography (DHPLC) analysis. Further study showed that the minimum TAIT for a successful MethyLight PCR reaction should be ≥ 9.4 ng (Ct_COL2A1_ ≤ 29.3), when the cutoff value of the methylated-*GFRA1* proportion for methylation-positive detection was set at 1.6%. After TAIT of the methylation non-informative frozen samples (*n* = 94; Ct_COL2A1_ > 29.3) was increased above the minimum TAIT, the methylation-positive rate increased from 72.3% to 95.7% for *GFRA1* and 26.6% to 54.3% for *P16*, respectively (*Ps* < 0.001). Similar results were observed in the FFPE samples. In conclusion, TAIT critically affects results of various PCR-based DNA methylation analyses. Characterization of the minimum TAIT for target CpG islands is essential to avoid false-negative results.

## INTRODUCTION

Alterations of DNA methylation play undisputed roles in cancer development through epigenetic inactivation and reactivation of tumor-related genes [1–8]. Methylation information on CpG islands are persistently maintained in various kinds of stored samples, i.e., genomic DNA extracted from frozen tissues, formalin fixed paraffin embedded (FFPE) tissues, cell line pellets, and various body fluids, which provide rich sample resources for DNA methylation studies and biomarker development. The methylation status of individual cytosines in genomic DNA can be quantitatively determined using bisulfite-treated DNA templates combined with different PCR-based assays including methylation-specific PCR (MSP), MethyLight, Pyrosequencing, denaturing high performance liquid chromatography (DHPLC), and matrix-assisted laser desorption/ionization time-of-flight mass spectrometry (MALDI-TOF MS) [9–15]. Most importantly, alterations of DNA methylation, even if they occur only in a very limited number of cells in specimens, can be sensitively detected using methylation or unmethylation specific assays. These DNA methylation changes can be used as one kind of optimal biomarker for predicting disease development and progression [16, 17].

It is well known that the efficiency of bisulfite-based DNA methylation PCR is generally much lower than regular PCR using double-strand genomic DNA as templates due to a number of reasons. This includes DNA degradation during bisulfite treatment, low stability of single-strand DNA, and strand-specific PCR amplification. Hence, more templates should be used in the bisulfite-PCR assays. Different amplification protocols recommend the use of 50–500 ng of bisulfite-treated DNA [14]. These strategies are not feasible for most researches when very limited amounts of DNA are available for PCR amplification, such as DNA samples extracted from endoscope biopsy, serum or plasma, and other body fluids. The variability in DNA methylation analysis may result from stochastic PCR amplification that often occurs at low template concentration and leads to false-negative detection [10]. Unfortunately, not enough attention has been paid to the false-negative events that may frequently occur when the amount of input template (TAIT) is lower than the detection limit, the minimum TAIT for a successful PCR reaction. Moreover, the exact impact of TAIT on outcomes of DNA methylation analysis has not been systematically studied. In the present study, we analyzed the influence of TAIT on the bisulfite-PCR-based DNA methylation analysis and provided a countermeasure to avoid the false-negative phenomenon.

## RESULTS

### Significant impact of TAIT on outcomes of DNA methylation analysis by MethyLight

**Figure 1:**
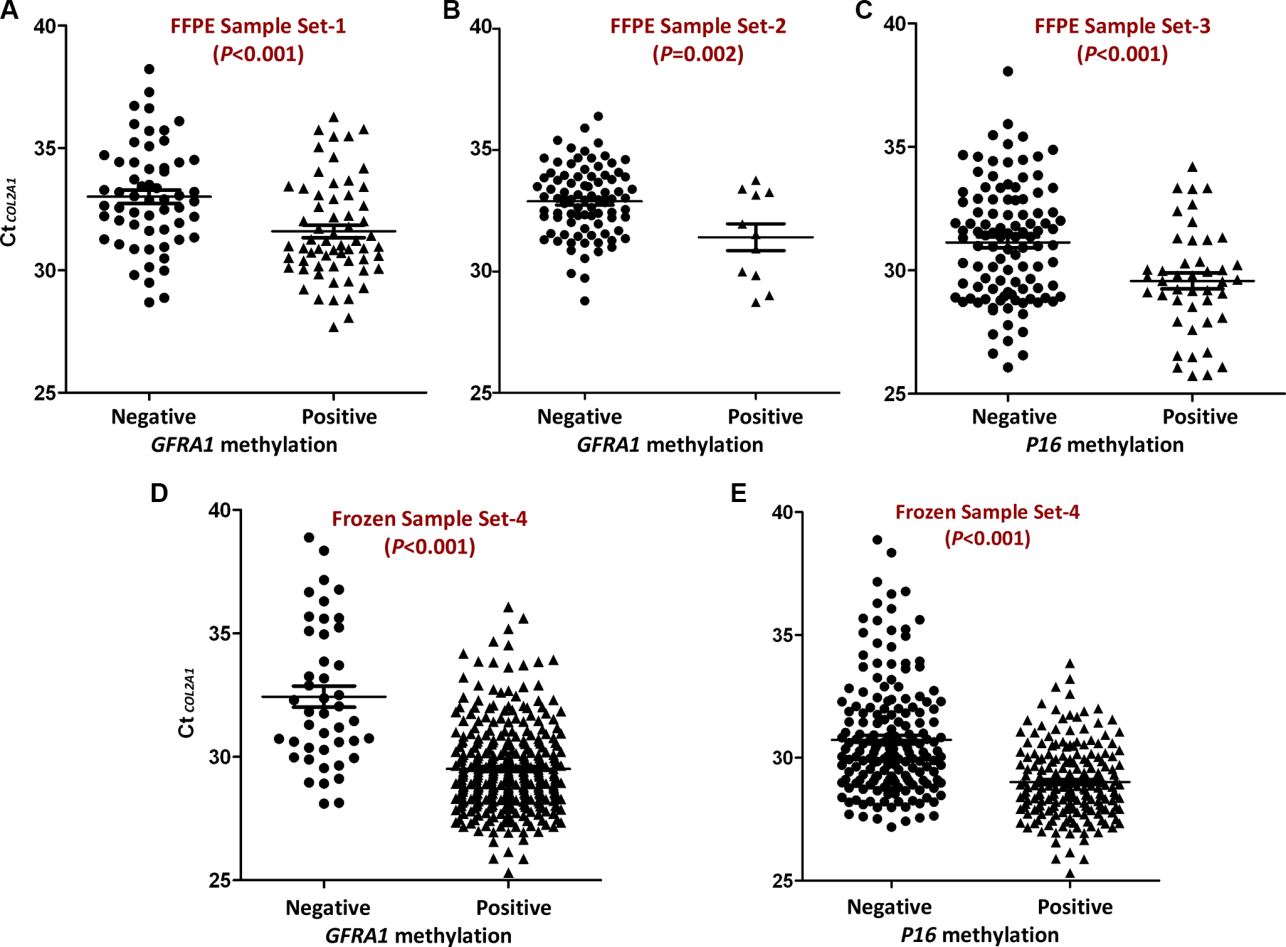
Comparison of Ct_COL2A1_ in *GFRA1* or *P16* methylation positive samples and methylation negative samples (**A** and **B**) The amount of input templates (TAIT) in surgical gastric carcinoma FFPE samples from Korean and Chinese, respectively. *GFRA1* methylation positive and negative: with or without methylation signal in MethyLight analysis; (**C**) TAIT in the oral mucosa biopsy FFPE samples with or without *P16* methylation signal in MethyLight analysis; (**D** and **E**) TAIT in surgical gastric carcinoma frozen samples with or without *GFRA1* or *P16* methylation signal in MethyLight analysis, respectively. The mean and SE values were marked with lines.

We used the average Ct value for the reference gene *COL2A1* as an indicator of TAIT level for a sample in MethyLight analysis. Through re-analyzing our previously published MethyLight data for FFPE surgical samples from Korean patients with gastric carcinoma (Set-1, Table 1) [5], we found that TAIT level in *GFRA1* methylation-positive samples (*n* = 62) was significantly higher than the methylation-negative ones (*n* = 58) (Ct_COL2A1_ [*median*]: 31.0 *vs*. 32.9, ΔCt = 1.9; *P* < 0.001; Figure 1A). To validate the TAIT difference, we analyzed another set of surgical gastric carcinoma FFPE samples (Set-2) using MethyLight, and observed the same phenomenon: the average TAIT level in *GFRA1* methylation-positive and -negative FFPE samples (*n* = 11 and 86) were 31.5 and 33.0, respectively (ΔCt = 1.5; *P* = 0.002, Figure 1B). Similarly, in oral mucosa biopsy FFPE samples (Set-3), TAIT level in *P16* methylation-positive oral biopsy FFPE samples (*n* = 44) was also much higher than the methylation-negative ones (*n* = 107) (29.6 *vs*. 31.4, ΔCt = 1.8; *P* < 0.001, Figure 1C). Unexpectedly, such differences were also found in DNA methylation analysis using frozen gastric surgical samples (Set-4): TAIT level in *GFRA1* methylation-positive samples (*n* = 332) was significantly higher than the methylation-negative ones (*n* = 44) (29.6 *vs*. 32.0, ΔCt = 2.4; *P* < 0.001, Figure 1D). Similarly, TAIT level in *P16* methylation-negative samples (*n* = 196) was lower than the methylation-positive ones (*n* = 180) either (30.5 *vs*. 29.1, ΔCt = 1.4; *P* < 0.001, Figure 1E). Together, these results indicate that TAIT level in the MethyLight assay may significantly affect results of DNA methylation detection. It is possible that false-negative results occur when the template molecules available for PCR amplification in tested samples are very low, or when TAIT level is lower than the minimum TAIT for a successful PCR reaction.

**Table 1:** Basic information for four sets of human tissue samples used in DNA methylation analysis

Set	Subjects	Storage	Organ	Pathological changes	*n*	Methylation in gene, assay	Proportion of Ct_COL2A1_≤ 29.3 samples^1^
Set-1	Korean	FFPE	Stomach	Adenocarcinoma	120	*GFRA1*, MethyLight	13.3%
Set-2	Chinese	FFPE	Stomach	Adenocarcinoma	97	*GFRA1*, MethyLight	3.1%
Set-3	Chinese	FFPE	Oral	Epithelial dysplasia	151	*P16*, MethyLight	33.8%
Set-4	Chinese	Frozen	Stomach	Adenocarcinoma or corresponding normal	376	*GFRA1 & P16*, MethyLight	75.5%
Set-5	Chinese	FFPE	Stomach	Epithelial dysplasia	116	*P16*, MSP	70.7%
Set-6	Chinese	Frozen	Colon	Adenocarcinoma or corresponding normal	185	*GFRA1*, DHPLC	90.8%

1 The value for the reference gene in MethyLight analysis without adjustment of input DNA.

It is well recognized that the quality of genomic DNA extracted from FFPE samples is lower than frozen samples because of DNA degradation in formalin fixating, paraffin embedding, and deparaffinage processes. This phenomenon is consistently displayed in the MethyLight analysis. The Ct_COL2A1_ value for most FFPE samples (Set-1, −2, and −3) was more than 29.3, the minimum TAIT value described below; in contrast, for most frozen samples (Set-4 and –6), ≤ 29.3 (Table 1, the last column). This indicates that the false-negative detection may frequently occur in MethyLight analysis using FFPE samples without adjustment of input DNA.

### Characterization of the minimum TAIT for successful MethyLight analyses

To characterize inference of unmethylated counterparts and the minimum amount (or detection limit) of methylated DNA templates for a success MethyLight reaction, a *GFRA1*-methylated template solution array was prepared through serial dilutions with *GFRA1*-unmethylated DNA. The bisulfite-treated DNA working solution (0.7 ng/μL) from RKO cells was initially diluted with another bisulfite-treated DNA stock solution (at equal concentration) from GES1 cells, and further diluted with TE buffer to various proportions (Table 2). 1.7 μL of these diluted template solutions was added into each MethyLight reaction (final volume, 15 μL; triplicate). Results showed that the Ct value for the methylated-*GFRA1* CpG islands (Ct_GFRA1_) gradually increased along with decrease in the amounts of DNA from RKO cells, whether diluted with DNA from GES1 cells or TE buffer. When the amount of the DNA from RKO cells was ≥ 0.15 ng per PCR reaction, the Ct_GFRA1_ value was consistently determined in the entire triplicate in the template array. Thus, 0.15 ng bisulfite-treated DNA is defined as the minimum amounts of template DNA from *GFRA1* methylated cells. Interestingly, along with increase in the amounts of *GFRA1*-unmethylated DNA from GES1 cells, the exact Ct_GFRA1_ values for the same amount of the DNA from RKO cells were not increased, indicating no interference resulted from the occurrence of unmethylated *GFRA1* templates. We also found that methylation signal for the methylated *P16* was also consistently detected when 0.15 ng bisulfite-treated DNA was added into each PCR reaction, suggesting a similar efficiency of PCR amplification for methylated *GFRA1* and *P16* templates.

**Table 2:** Ct values for the input reference *COL2A1* and *GFRA1* in various bisulfite-treated DNA samples from RKO cells serially diluted with TE buffer and/or bisulfite-treated DNA from GES1 cells in MethyLight analysis

RKO-DNA diluted by		Total amounts of input DNA from RKO & GES1		Net amounts of DNA templates from RKO cells			
GES1-DNA	TE-Buffer	ng/reaction	Ct_COL2A1_	ng/reaction	Percentage^a^	Ct_GFRA1_	Ct_P16_
–	–	18.78	27.90	1.18	6.3%	**32.82**	**33.58**
1/2	–	18.78	28.18	0.59	3.1%	**33.92**	**35.04**
1/4	–	18.78	27.93	0.29	1.6%	**34.78**	**35.13**
1/8	–	18.78	28.18	0.15	0.8%	**35.84**	**36.61**
1/16	–	18.78	28.09	0.07	0.4%	37.38^1^	**37.76**
1/32	–	18.78	28.05	0.04	0.2%	38.25^1^	38.49^1^
1/64	–	18.78	28.10	0.02	0.1%	38.99^1^	38.87^1^
–	1/2	9.39	29.34	0.59	6.3%	**34.16**	**34.70**
1/2	1/2	9.39	29.35	0.29	3.1%	**35.35**	**36.21**
1/4	1/2	9.39	29.33	0.15	1.6%	**36.15**	**37.30**
1/8	1/2	9.39	29.41	0.07	0.8%	37.41^1^	**38.07**
1/16	1/2	9.39	29.53	0.04	0.4%	39.84	**38.63**
1/32	1/2	9.39	29.38	0.02	0.2%	37.95^1^	38.74^1^
1/64	1/2	9.39	29.26	0.01	0.1%	36.83^1^	Undet^2^
–	1/4	4.70	30.73	0.29	6.3%	**35.28**	**36.78**
1/2	1/4	4.70	31.08	0.15	3.1%	**37.59**	**37.73**
1/4	1/4	4.70	31.10	0.07	1.6%	39.17	38.18^1^
1/8	1/4	4.70	30.64	0.04	0.8%	38.12^1^	37.71^1^
1/16	1/4	4.70	30.95	0.02	0.4%	39.74^1^	Undet^2^
1/32	1/4	4.70	30.78	0.01	0.2%	Undet^2^	Undet^2^
1/64	1/4	4.70	30.83	0.005	0.1%	37.96	Undet^2^
–	1/8	2.35	31.61	0.15	6.3%	**36.67**	**37.10**
1/2	1/8	2.35	32.04	0.07	3.1%	37.45^1^	38.10^1^
1/4	1/8	2.35	31.93	0.04	1.6%	38.14	38.48^1^
1/8	1/8	2.35	31.81	0.02	0.8%	37.96^1^	Undet^2^
1/16	1/8	2.35	31.92	0.01	0.4%	41.02^1^	38.93^1^
1/32	1/8	2.35	31.64	0.005	0.2%	Undet^2^	Undet^2^
1/64	1/8	2.35	31.62	0.002	0.1%	Undet^2^	Undet^2^

The underlined values indicate the total amount of input DNA, Ct_COL2A1/GFRA1/P16_ when the minimum net amount of RKO cell DNA (0.15 ng/reaction) was added into each MethyLight reaction; The values in red characters are various Ct values when 0.15 ng of RKO DNA was presented in 9.39 ng of input DNA (1.6%) in each MethyLight reaction. Ct value is written in bold characters when informative methylation signal is always determined in triplicate.

1 Ct_GFRA1_ value is undetermined in one or two tubes in triplicate.

2 Ct_GFRA1_ value is undetermined in all of triplicate.

### Strategy to avoid false negative results in DNA methylation analysis

Generally, both methylated and unmethylated alleles exist in human tissues due to the heterogeneous cell composition. Despite imprinting of genes, the proportion of methylated alleles for CpG islands in disease-related genes in tissues may greatly change as adaptations to various environmental factors occur. This is particularly true, especially in biopsies from pathological lesions comparing with normal tissues. Therefore, characterization of the minimum amount of total input genomic DNA for a successful methylation analysis is a critical issue to avoid false negative results. According to results from the above minimum TAIT for *GFRA1* and *P16* methylation analyses, when the cutoff value for the proportion of methylated *GFRA1* or *P16* alleles in a tested sample is set at 1.6%, in order to avoid false negative results, at least 9.4 ng bisulfite-treated input DNA (Ct_COL2A1_ ≤ 29.3) should be added into each PCR reaction. To evaluate the false negative rate in the methylation non-informative samples (Ct_COL2A1_ > 29.3) in previous methylation analysis, we re-analyzed the methylation status of the representative genes using increased TAIT (Ct_COL2A1_ ≤ 29.3) as described in details below.

**Figure 2:**
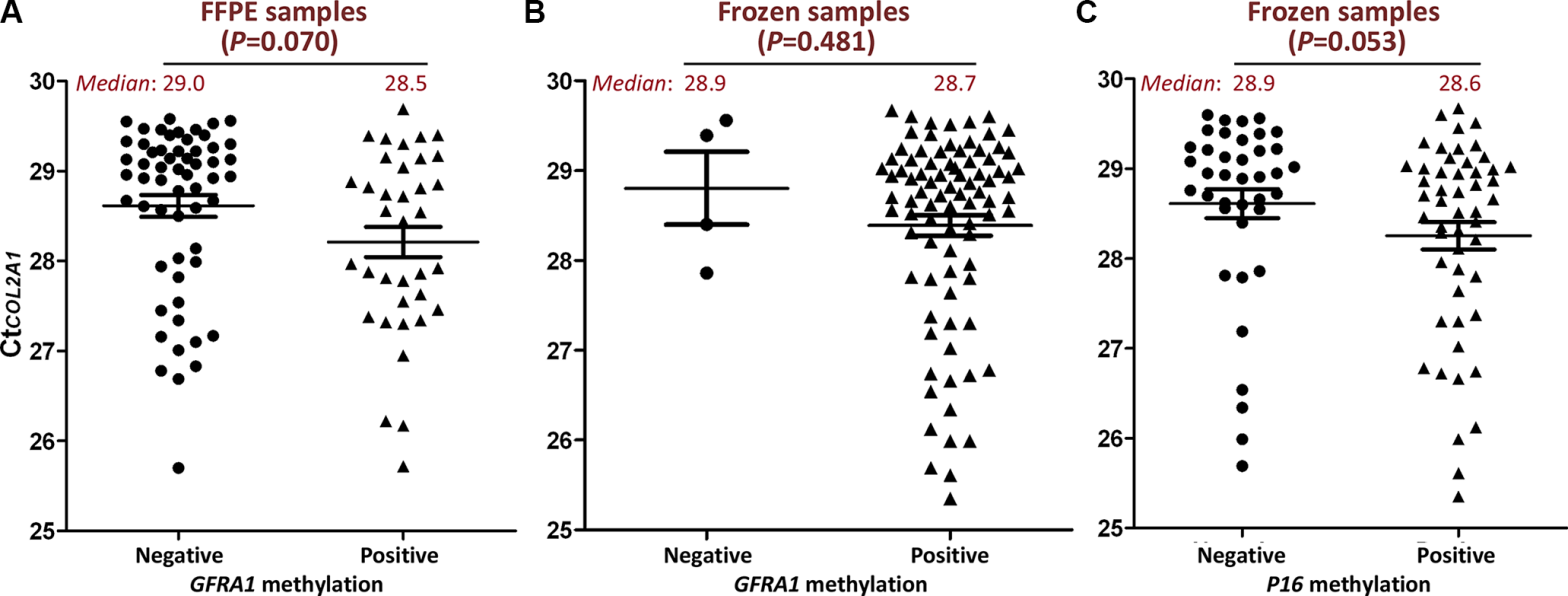
Comparison of Ct_COL2A1_ in *GFRA1* or *P16* methylation positive re-analyzed samples and methylation negative ones The amount of input templates (TAIT) in each re-analyzed sample was increased to above the minimum TAIT (Ct_*COL2A1*_ ≤ 29.3) according to the Ct_*COL2A*_ value obtained in the first-round analysis illustrated in Figure 1. (**A** and **B**) TAIT in surgical gastric carcinoma FFPE and frozen samples, respectively. *GFRA1* methylation positive and negative: with or without methylation signal in MethyLight analysis; (**C**) TAIT in surgical gastric carcinoma frozen samples with or without *P16* methylation signal in MethyLight analysis. The mean and SE values were marked with lines. The median values were also inserted.

Among Set-2 and Set-4, 96.9% (94/97) FFPE samples and 25.0% (94/376) frozen samples were detected with Ct_COL2A1_ > 29.3 (Table 1, the last column), suggesting the *GFRA1* methylation status may be underestimated due to lower TAIT level. Therefore, the methylation status of *GFRA1* in these samples was re-analyzed using more input template (Ct_COL2A1_ ≤ 29.3) and MethyLight assay. Results revealed that the *GFRA1* methylation-positive rate was significantly increased from 10.6% to 39.4% (*P* < 0.001) for these 94 FFPE samples. For the 94 frozen samples, the *GFRA1* methylation-positive rate also significantly increased from 72.3% to 95.7% (*P* < 0.001). Similarly, *P16* methylation-positive rate increased from 26.6% to 54.3% (*P* < 0.001). Subsequently, no significant difference of TAIT levels was found between the methylation–positive and –negative samples in the re-analysis. The average TAIT level in the *GFRA1* methylation-positive FFPE samples (*n* = 37) was not significantly higher than the methylation-negative ones (*n* = 57) (Ct_COL2A1_ [*median*]: 28.5 *vs*. 29.0, ΔCt = 0.5; *P* = 0.070; Figure 2A), and the average TAIT level in the *GFRA1* or *P16* methylation-positive frozen samples (*n* = 90 or 51) was similar with their methylation-negative samples (*n* = 4 or 43) (Ct_COL2A1_ [*median*]: 28.7 *vs*. 28.9, ΔCt = 0.2, *P* = 0.481 for *GFRA1*, Figure 2B; 28.6 *vs*. 28.9, ΔCt = 0.3, *P* = 0.053 for *P16*, Figure 2C).

### Evaluation of false negative prevalence in other PCR-based methylation assays

We wonder whether TAIT level also affects results of other PCR-based methylation assays. The amounts of template DNA in Set-5 and Set-6 samples (Table 1) were detected using MethyLight at first. The *P16* and *GFRA1* methylation were analyzed using MSP and DHPLC, respectively. Because the reported detection limit (0.1–0.4%) for MSP and DHPLC assays [13, 18] was similar to MethyLight (Table 1), we directly used the minimum TAIT value for MethyLight to classify the methylation informative and not informative samples in MSP and DHPLC analysis. For 34 of 116 FFPE samples (29.3%) in Set-5, TAIT values in MSP analysis were lesser than the minimum TAIT (Ct_COL2A1_ > 29.3). The *P16* methylation-positive rate in 82 informative samples (Ct_COL2A1_ ≤ 29.3) was significantly higher than that in the 34 not informative samples (Ct_COL2A1_ > 29.3) (78.0% vs. 47.1%, *P* < 0.001). Similarly, for 17 of 185 frozen samples (9.2%) in Set-6, TAIT values in the PCR amplification step in DHPLC analysis were less than the minimum TAIT (Ct_COL2A1_ > 29.3). The *GFRA1* methylation-positive rate in 168 informative samples was also higher than that in the not informative samples (58.3% vs. 41.2%), but not significant (*P* = 0.174). These results indicate false negative results do occur when the amount of input DNA in MSP or DHPLC analysis is less than the minimum value.

## DISCUSSION

Various PCR assays for DNA methylation analysis have been developed using bisulfite-treated genomic DNA as PCR template [9–15]. Poor quality and low quantity of input DNA are always important parameters to decrease PCR efficiency that may lead to methylation non-informative (false negative) result. This is becoming a serious problem as more researchers become interested in DNA methylation in both basic and translational research. In the present study, we have systematically evaluated the exact influence of TAIT on the detection of the methylation status of CpG islands in two representative genes *GFRA1* and *P16* using MethyLight assay, and found that TAIT is a critical factor that leads to a relative proportion of false negative results, especially in FFPE samples. Meanwhile, we have also provided a strategy to avoid the false negative results through determining the minimum TAIT value (detection limitation) and using more input DNA than the minimum TAIT.

It has previously been reported that the number of molecules available for PCR amplification is critical for accurate characterization of methylation ratios using MALDI-TOF MS assay [15]. When few molecules are present, the reaction can still be performed successfully, but the sampling error has a devastating effect on the confidence level of the quantitative results in a representative sample. Unfortunately, enough attention has still not been paid to the TAIT-related false negative in most of studies. In one of our recent works, we used the minimum TAIT strategy to avoid false negative results in *P16* methylation analysis in a multicenter prospective study [2]. Here, we have described for the first time the impact of TAIT on outcomes of DNA methylation analysis and describe how to overcome this obstacle in details.

We have not only observed the impact of TAIT on outcome of DNA methylation analysis using MethyLight assay, but also observed the same phenomenon using MSP and DHPLC assay. The TAIT-related inference might be applicable for other PCR-based methylation assays such as Pyrosequencing and COBRA. The remarkable effect of the number of input templates available for PCR amplification in MALDI-TOF MS assay supports this extrapolation [15]. The amount of input DNA for only 17 of 185 Set-6 samples was lesser than the minimum TAIT in the PCR amplification step in DHPLC analysis. This may account for that the difference of *GFRA1* methylation positive rate between the informative and not informative samples was not significant.

PCR amplification efficiency is amplicon sequence-dependent. Although we found the minimum TAIT level for *GFRA1* was not different from *P16*, we recommend determining the TAIT value for each interested amplicon in CpG islands, especially for these genes with different copy number in the genome. Because commercial standards for methylated and unmethylated DNA substances are not available at present, we have to use the genomic DNA from cancer cell lines as the homogenously methylated and unmethylated reference DNA to obtain standard curves in MethyLight analysis. Application of artificially and completely methylated genomic DNA sample by *M.SssI* DNA methyltransferase may be an alternative. Although the artificial DNA sample cannot be prepared as fresh or FFPE tissue controls, we recommend regularly using standard methylated and unmethylated DNA controls during DNA extraction, purification, bisulfite modification, and PCR processes to monitor the recovery, conversion, and amplification rates.

The minimum TAIT value may be different from the cutoff value in clinical practices. Clinical cancer tissues are mixtures of tumor cells and normal cells. A tumor may contain different cell sub-populations with various genetic and epigenetic changes. For a sample composed of high fraction of cancer cells with homogenous target DNA methylation changes (target cells), the minimum TAIT value may be lower than a sample composed of low fraction of target cells. Although the absolute amount of DNA template is the same for different kinds of samples, but the cutoff value for the proportion of methylated CpG islands may be sample- or gene-dependent. For example, in one of our prospective studies to predict malignant transformation risk of precancerous lesion epithelial dysplasia, the cutoff value for the proportion of *P16*-methylated cells was set at 1.6% to define *P16* methylation-positive samples and avoid false negative detections [2]. In another study on gastric cancer metastasis, *GFRA1* methylation signal was detected in 60.2% cancer tissue samples from the discovery cohort, a cutoff value for the proportion of *GFRA1*-methylated cells (> 26.4%) was further calculated according to ROC curve to define *GFRA1* methylation-positive samples in the Kaplan-Meier survival analysis [5]. The minimum TAIT value can be used to avoid false negative detection; the cutoff value can be used to subclass samples with different prognosis or therapy sensitivity.

The minimum TAIT value was calculated based on both the absolute amount of genomic DNA containing fully methylated target alleles in input template and the total amounts of available templates in most frozen samples at good quality. The *P16* methylation signal could be consistently determined when 0.15 ng (or more) *P16-*methylated DNA was added into each PCR reaction. In order to enable most DNA samples methylation-informative after adjustment of input DNA, we selected 1.6% as the cutoff proportion of methylated *P16* to definite a *P16* methylation-positive sample (the minimum required total DNA, 38 ng; 9.4 ng/MethyLight reaction, two reactions for methylated-*P16* and two reactions for the *COL2A1* reference). When the clinical cutoff value is much higher than the detection limitation (i.e. *GFRA1* methylation), the minimum TAIT value could be reduced further.

Except to increase the amount of input DNA, a quantitative nested approach (if available) may be an alternative way to avoid false negative detection. However, a proper negative control should be used to avoid false-positive detection, which often occurs in qualitative nested analyses. For example, one can use a DNA sample containing < 1.6% methylated*-P16* as a negative control if the cutoff proportion for the methylation-positive is set at 1.6%.

In addition, because the Ct_COL2A1_ value not only represents the amount of input templates, but also represents the quality of input DNA and efficiency of bisulfite conversion of unmethylated cytosine residues, we recommend using the Ct_COL2A1_ value as an ideal TAIT reference to avoid false-negative events.

In conclusion, TAIT significantly impacts on outcomes of DNA methylation analysis using PCR-based assays. The minimum TAIT should be determined for each amplicon sequence in genomic DNA extracted from different kinds of samples to avoid false negative results.

## MATERIALS AND METHODS

### Cell line and culture

*GFRA1* and *P16* fully methylated cell line RKO and unmethylated cell line GES1 were kindly provided by Dr. Guoren Deng at University of California San Francisco and Dr. Yang Ke at Peking University Cancer Hospital/Institute respectively. Cell lines were identified by STR analysis before use [5]. Cells were cultured in RPMI-1640 and 10% FBS.

### Human gastric mucosa samples (Table 1)

Set-1 and Set-2 gastric carcinoma FFPE samples consist of 120 specimens from Seoul National University Hospital and 97 specimens from Peking University Beijing Cancer Hospital, respectively. For Korean sample Set-1, *GFRA1* methylation data has been published in [5]. Set-3 samples consist of 151 oral epithelial dysplasia FFPE samples from Peking University School of Stomatology, Capital Medical University School of Stomatology, and Fourth Military Medical University Hospital of Stomatology collected between 2009 and 2011 in a fully anonymized and de-identified form, which were part of samples used in a multicentre prospective study (trial number NCT01695018, available at http://ClinicalTrials.gov). For those samples, *P16* methylation data has been published in [2]. Set-4 frozen primary gastric carcinoma surgical tissues and their corresponding surgical margin samples (*n* = 376) were from 188 patients who underwent surgical treatment at Peking University Cancer Hospital & Institute. These 188 patients were recruited in an ongoing prospective study (trial number NCT02159399, available at http://ClinicalTrials.gov). Set-5 samples consist of 116 gastric epithelial dysplasia FFPE samples from Peking University Beijing Cancer Hospital. Set-6 samples consist of 185 frozen colon adenocarcinoma samples from the same Hospital. These studies were approved by the local Institution Review Boards (IRB) at Peking University Beijing Cancer Hospital covered the collection and research use of tissues from all sites. All patients were given written informed consent unless the IRB permitted a waiver.

### DNA extraction and bisulfite modification

Genomic DNA was extracted from cell line and frozen tissue samples using phenol/chloroform method. Briefly, paraffin-embedded tissues were treated with xylene and digested with proteinase K to obtain DNA [19]. The unmethylated-cytosine bases in the genomic DNA were converted to uracil bases by addition of 5 mol/L of sodium bisulfite at 50°C overnight [20].

### Quantification of DNA

A Nanodrop ND-1000 spectral photometer (Nanodrop Technologies, Wilmington, DE, USA) was used to detect DNA concentration. For calculation of the DNA concentration a multiplication factor of 33 was used for single-stranded DNA (bisulfite-DNA), and 50 for double-stranded DNA (genomic DNA).

### Preparation of methylated bisulfite-DNA solution serials

Stock solution of bisulfite-DNA from RKO cells (10 ng/μL) was serially diluted at different ratios (1:0, 1:1, 1:3, 1:7, 1:15, 1:31, and 1:63) through adding stock solution of bisulfite-DNA from GES1 cells (10 ng/mL). These solutions were further diluted with TE buffer at ratios 1:0, 1:1, 1:3, and 1:7 to prepare various working solutions. All of these working solutions were used in MethyLight assay in triplicate through adding 1.7 μL working solution into PCR mix (total volume 15 μL).

### MethyLight

MethyLight assay was performed as previously described to detect the proportion of methylated CpG islands within *P16* (115bp) and *GFRA1* (158bp), respectively [2, 5]. Gene-specific probes labeled with 6FAM and TAMRA were employed to quantify the relative copy number of methylated alleles compared to the *COL2A1* reference (91 bp) [21]. When methylation signal for target CpG islands was consistently detected, it was defined as methylation-positive (without use of cutoff value). The average Ct value for the *COL2A1* reference was used as an indicator of the TAIT level for a sample.

### Methylation-specific PCR (MSP)

The 150 bp methylated *P16* amplicon was analyzed used in the regular MSP analysis as described previously [22, 23].

### Denatured high performance liquid chromatography (DHPLC)

The 522 bp methylated *GFRA1* amplicon was analyzed used DHPLC and fluorescence detector as described previously [5].

### Statistical analysis

Pearson's Chi-square test was used to compare categorical variables. The Mann-Whitney *U*-test and Student's *t*-test were used to compare the Ct value of *COL2A1* (Ct_COL2A1_) between methylation-positive and -negative samples. All statistical tests were two-sided, and *P* < 0.05 was considered statistically significant.
